# Biomechanical effects of fixed-bearing femoral prostheses with different coronal positions in medial unicompartmental knee arthroplasty

**DOI:** 10.1186/s13018-022-03037-0

**Published:** 2022-03-09

**Authors:** Pengcheng Ma, Aikeremujiang Muheremu, Siping Zhang, Qian Zheng, Wei Wang, Kan Jiang

**Affiliations:** 1 Department of Orthopedics, Sixth Affiliated Hospital of Xinjiang Medical University, 39 Wuxing Nan Rd, Tianshan District, Ürümqi, 830001 Xinjiang People’s Republic of China; 2 Department of Spine Surgery, Sixth Affiliated Hospital of Xinjiang Medical University, Ürümqi, 830001 Xinjiang People’s Republic of China; 3 Urumqi DW Innovation InfoTech Co., Ltd., Ürümqi, 830001 Xinjiang People’s Republic of China

**Keywords:** Knee, Unicompartmental knee arthroplasty, Prosthesis fitting, Biomechanics, Stress, Finite element analysis

## Abstract

**Background:**

To study the biomechanical effects of femoral prostheses at different coronal positions using finite element analysis and provide a clinical reference for unicompartmental knee arthroplasty (UKA).

**Methods:**

A normal knee joint model was established and verified, establishing 13 working conditions for the femoral prosthesis: the standard position, varus and valgus angles of 3°, 6° and 9° and medial and lateral translations of 1 mm, 3 mm and 5 mm. The stress changes at different positions were analysed, including the polyethylene (PE) insert upper surface, the surface of lateral compartment cartilage and the surface of cancellous bone under tibial prosthesis.

**Results:**

The stresses on the PE insert upper surface and the cancellous bone surface increased with increasing femoral prosthesis valgus/varus, and the stress increased gradually during medial to lateral translation. The stress change is more significant during valgus and lateral translation. However, the stress on the cartilage surface decreases in the process of varus to valgus and medial translation to lateral translation.

**Conclusion:**

The fixed-bearing femoral prosthesis of the medial UKA should avoid translation or varus/valgus tilt on the coronal plane as much as possible. The obvious misalignment of the femoral prosthesis will significantly affect the stress on the internal structure of the knee joint, especially the PE insert and cartilage surface. A femoral prosthesis coronal tilt of more than 6° may significantly increase the stress on the PE surface, and varus of more than 6° may significantly increase the stress on the cartilage surface. For the femoral prosthesis position at the distal end of the femoral condyle, it is recommended to be placed in the centre.

## Background

The knee is the most susceptible joint to osteoarthritis. There are more than 250 million knee osteoarthritis (KOA) patients around the world, accounting for nearly 40% of people over the age of 60 [1, 2]. For KOA, approximately 33% of patients only have degeneration of the medial compartment of the knee joint, with the rest of the compartments being relatively normal [3]. This contributes to the development of UKA. Unlike total knee arthroplasty (TKA), UKA retains the healthy lateral compartment and the inherent soft tissue of the knee joint, leading to less trauma and quick recovery [4].

Although UKA has been an option for the treatment of terminal medial knee compartment osteoarthritis, its application is still limited [5]. The low survival rate of prostheses is the most prominent problem that restricts the applications of UKA in orthopaedic clinics. Studies have shown that the 10-year survival rate of UKA prostheses is generally 80–85% and lower than that of TKA prostheses [5]. Postoperative complications, such as loosening of the prosthesis, wear of the PE insert and progression of contralateral compartment arthritis, are the main reasons leading to the low survival rates of prostheses [6]. The occurrence of complications is closely related to prosthesis malalignment. Because a large number of anatomical structures remain, it is of great significance to strengthen the matching degree between the prosthesis and the surrounding tissue when the line of force is restored during the operation. The fault tolerance rate of UKA prostheses is less than that of TKA, and a minor prosthesis malalignment may lead to abnormal changes in the internal stress of the knee joint, thus accelerating the wear of cartilage and PE inserts and eventually causing prosthesis loosening [7, 8].

The probability of prosthesis malalignment and the degree of stress change after malalignment are associated with the type of prosthesis. In the process of positioning the femoral prosthesis, the fixed-bearing prosthesis is more prone to deviating than the mobile-bearing prosthesis due to the lack of special osteotomy positioning equipment [9, 10]. The stress change caused by the malalignment of the fixed-bearing prosthesis is theoretically greater than that of the mobile-bearing prosthesis with a higher requirement for positioning accuracy [11]. The result of a finite element analysis showed that the stress is higher for the fixed-bearing prosthesis on the upper surface of the PE insert and the cartilage surface of the contralateral compartment compared to those of the mobile-bearing prosthesis [12].

Currently, there are few finite element studies researching the placement position of fixed-bearing femoral prostheses. Moreover, the stress change trend of each knee joint structure at different femoral prosthesis positions remains unknown [8, 13].

To further study the biomechanical effects caused by the placement of femoral prostheses at different positions, finite element analysis was used in this study, which can directly show the stress value and the distribution [14]. Several UKA models of the femoral prosthesis have been established with different varus/valgus and translation on the coronal plane. To explore the safe range of femoral prosthesis placement, this study observes the effects of femoral prosthesis position change on the following three aspects: (1) the surface stress on the upper surface of the PE insert, (2) the surface stress on the cancellous bone under the tibial prosthesis, and (3) the surface stress on the cartilage of the lateral compartment.

## Materials and methods

### Establishment of a normal knee joint model

Imaging data were obtained from a 35-year-old female volunteer who had no history of skeletal muscle disease. There were no abnormal findings on right knee anteroposterior and lateral radiographs and 1:1 full-length standing radiographs of both lower limbs, symmetrical bilateral tibiofemoral joint gaps, and no noticeable pathological changes or developmental abnormalities in the bone and joint. A 64-row computed tomography (CT) scanner (Siemens, Germany) with a 0.6 mm layer thickness was used to perform a full-length plain scan of both lower extremities. A 3.0T magnetic resonance imaging (MRI) scanner (Siemens, Germany) was used to perform a sagittal plane scan of the right knee with a layer thickness of 1 mm, covering 15 cm each of the distal femur and proximal tibia.

**Fig. 1:**
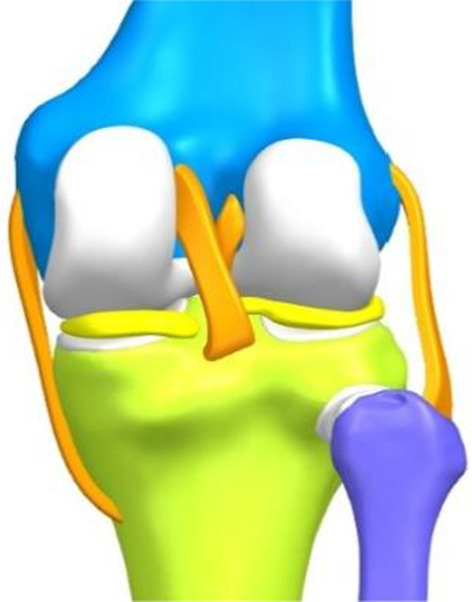
Complete knee model

The images were imported into Mimics 20 (Materialize, Belgium) to create masks of the knee bones, cartilage and soft tissues, such as ligaments and meniscus. After the 3D reconstruction, the masks were smoothed, and all the masks were aligned to form a complete lower limb model. Moreover, the lateral angle of the distal femur was measured at approximately 82°, and the medial angle of the proximal tibia was measured at approximately 91°. Both values were within the normal range, and the area 15 cm above and below the centre of the right knee was selected. The structure models were noise-smoothed and surface-fitted in Geomagic Studio 2014 (3D Systems, America) and stored as.IGES files. Afterwards, NX 1911 (Siemens, Germany) was imported to build a solid model of each structure, assembled into a complete knee model (Fig. 1), optimized, and saved as an.STP file. The.STP file was imported into Ansys Workbench 2019 (ANSYS, America) for meshing, material property assignment, and boundary condition setting. All structures were meshed with 10-node 4-sided cells, and convergence was verified afterwards, with convergence defined as less than 5% variation between two adjacent meshes. According to previous studies, the ligament was defined as a hyperelastic isotropic material and represented by the neo-Hooke model [15–17]. The rest of the structures were defined as linear elastic isotropic materials and assigned parameters (Table 1). Six contact pairs of femoral and tibial cartilage, tibial cartilage and meniscus, and femoral cartilage and meniscus were established. Using the penalty function algorithm [18], the bone to ligament, bone to cartilage and tibial cartilage to the meniscus are defined as bound connections, and the femoral cartilage to the meniscus and femoral cartilage to tibial cartilage are defined as frictionless finite sliding surface contacts. This contact ensures that the stability of the knee joint is provided by the equilibrium state between the structures rather than by friction [18]. The boundary conditions were set to the relative extension position of the femur and tibia, and only the femoral flexion and extension activities were constrained, while the activities in other directions were not constrained. The distal tibia and fibula were constrained entirely [19] to form a normal knee model.

**Table 1:** Material properties of different structures

Structures	Young's modulus (MPa)	Poisson's ratio
Cortical bone	17,000	0.3
Cancellous bone	350	0.25
Menisci	27.5	0.33
Cartilage	15	0.46
Cobalt-chromium-molybdenum alloy	210,000	0.29
Polyethylene (PE)	850	0.4
Methacrylate	1940	0.4

### Establishment of UKA models with different femoral prosthesis positions

**Fig. 2:**
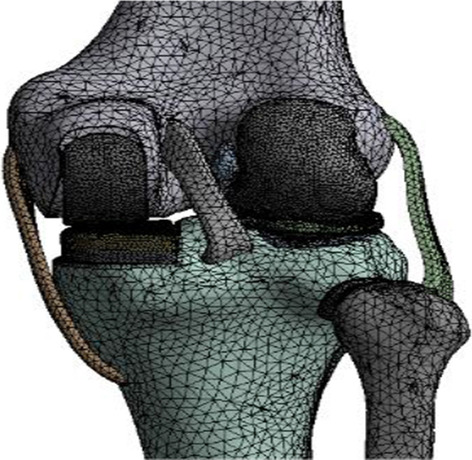
The finite element model of UKA in the standard position of the prosthesis

A Link-Sled® fixed-bearing prosthesis (LINK, Germany) of the appropriate size was selected and scanned with a FreeScanX5 3D scanner (Tianyuan 3D Technology Co, China). The solid model of the prosthesis after trimming and smoothing was imported into Ansys Workbench 2019. The meshing was performed with 4-sided cells, the metal part was cobalt-chromium-molybdenum alloy, and the linear part was ultrahigh-molecular-weight PE, both defined as linear-elastic isotropic materials and assigned values according to previous studies [17] (Table 1). The medial UKA of the knee was simulated using Boolean operations, with the tibial prosthesis covering the maximum tibial osteotomy surface with a posterior tilt angle of 5°, no varus or valgus, no axial rotation and the height of the joint line after installation of the prosthesis was the same as before the osteotomy. The standard position of the femoral prosthesis was to cover the centre of the medial femoral condyle and to cover the distal end of the posterior femoral condyle as much as possible, perpendicular to the tibial prosthesis in the coronal plane and without axial rotation [14]. A 1 mm methyl methacrylate bone cement filling was simulated between the prosthesis and the osteotomy surface and defined as a linearly elastic isotropic material (Table 1), forming a finite element model of UKA in the standard position of the femoral prosthesis (Fig. 2).

**Fig. 3:**
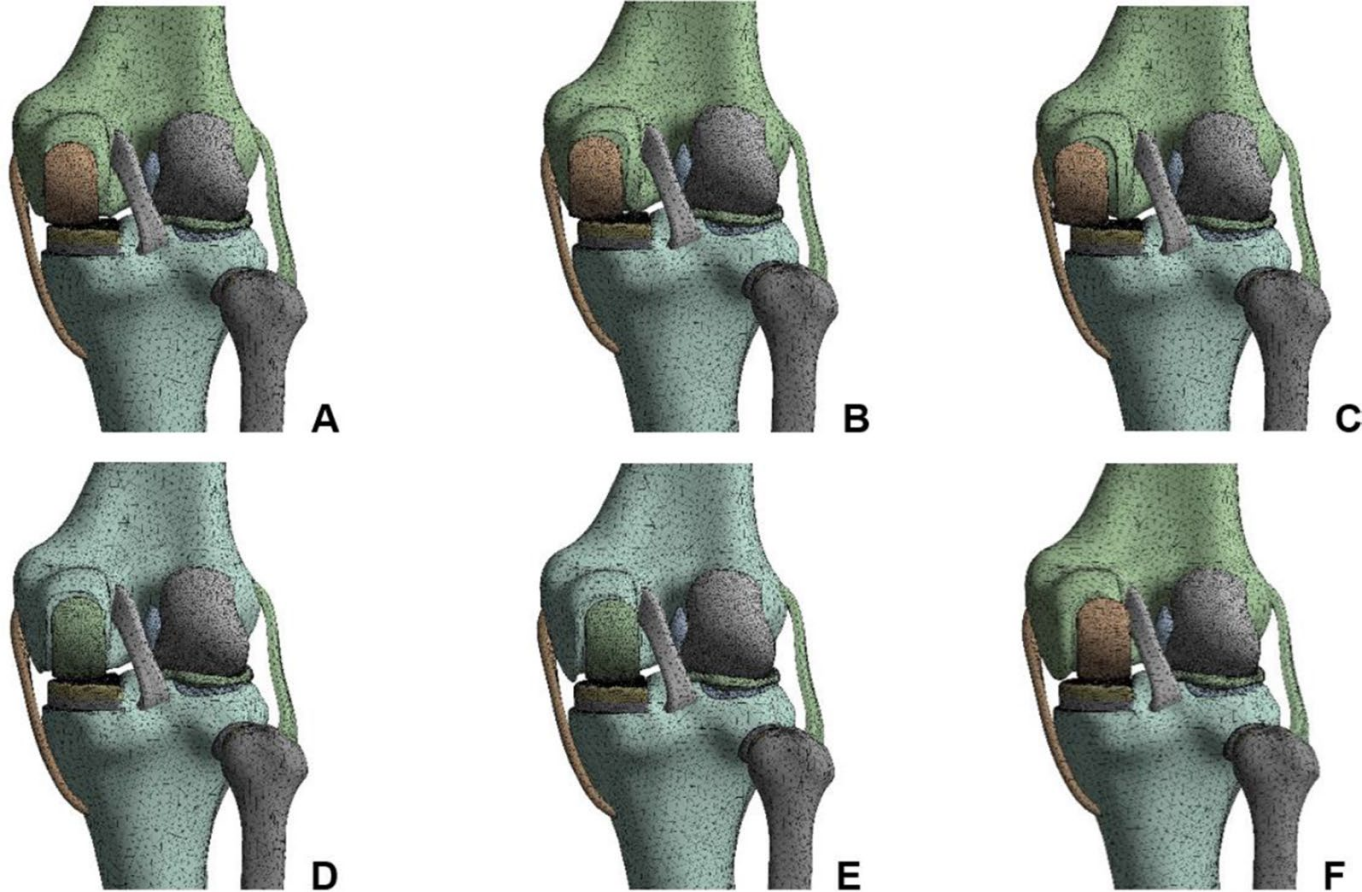
Finite element models of UKA with different translations of the femoral prosthesis. **A** 1 mm medial, **B** 3 mm medial, **C** 5 mm medial, **D** 1 mm lateral, **E** 3 mm lateral, **F** 5 mm lateral

The standard position of the femoral prosthesis was used as the reference. The UKA models were established as varus/valgus 3°, 6°, 9° of femoral prosthesis, 1 mm, 3 mm and 5 mm of medial/lateral translation (Fig. 3), for a total of 12 positions. The femoral prosthesis and the upper surface of the PE insert were set as frictionless limited sliding contact, the osteotomy surface-bone cement-prosthesis were all bound contacts, and the contact relationships of other structures were the same as the normal knee model [19]. The boundary conditions were set to the relative extension position of the femur and tibia, and the femur was allowed merely to perform varus/valgus, anterior/posterior translation and vertical activities. The distal tibiofibular region was completely restrained [20].

### Stress loading and model validation

All UKA models were loaded with the same stress loading method, and a load of 1000 N along the mechanical axis of the femur downwards was applied to the distal femur. The boundary conditions of each model were kept constant during the loading process, and convergence was verified after loading [17].

Two methods were used to validate the normal knee joint model [21]. Method 1: A 1000 N load was applied downwards along the femoral mechanical axis to observe the distribution of cartilage and meniscal equivalent forces in the medial and lateral compartments of the knee joint, the peak value, and the load ratio. Method 2: Simulated anterior drawer experiment, changing the boundary conditions to restrain only the flexion activity of the tibiofibular axis and completely restrain the femur activity, applying a 134 N anterior load perpendicular to the coronal plane across the midpoint of the line between the medial and lateral margins of the tibial plateau, and observing the displacement distance. The results of the two methods of validation were compared with the results of previous studies, and the model was considered reasonable if the differences were small.

### Statistical analysis

The high-stress values were expressed as the mean of the first 6 points with the highest stress value, and the data were analysed using SPSS 25.0 software (IBMSPSS, Chicago, IL). The high-stress values are measures that satisfy a normal distribution and are expressed as the mean ± standard ($\overline{x}\, \pm \,S$) deviation. The high-stress values at each site in each UKA model were compared with those in the UKA model for the standard position of the prosthesis using an independent samples *t* test, with *P* < 0.05 indicating a statistically significant difference.

## Results

### Results of the model validation

**Fig. 4:**
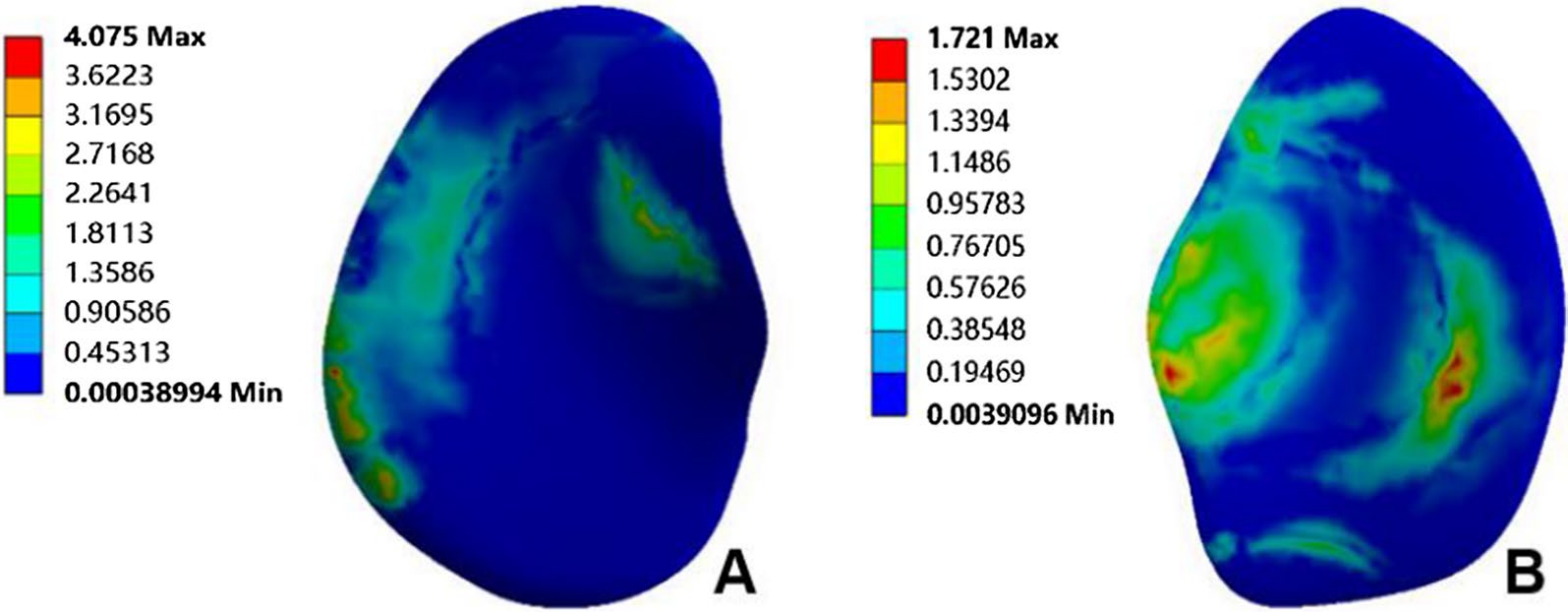
Peak value and distribution of equivalent stress on the surface of medial and lateral tibial cartilage. **A** Medial tibial cartilage, **B** lateral tibial cartilage

**Fig. 5:**
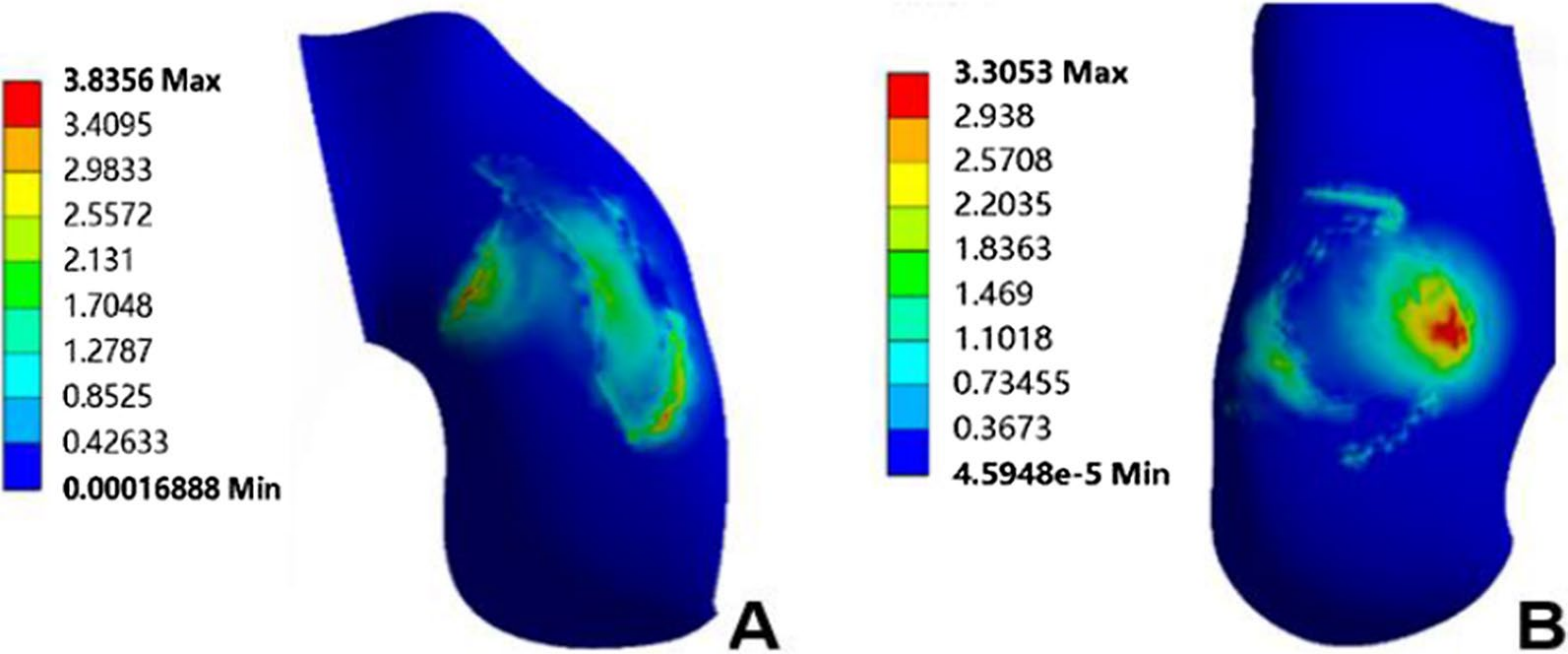
Peak value and distribution of equivalent stress on the surface of medial and lateral femoral cartilage. **A** Medial femoral cartilage, **B** lateral femoral cartilage

**Fig. 6:**
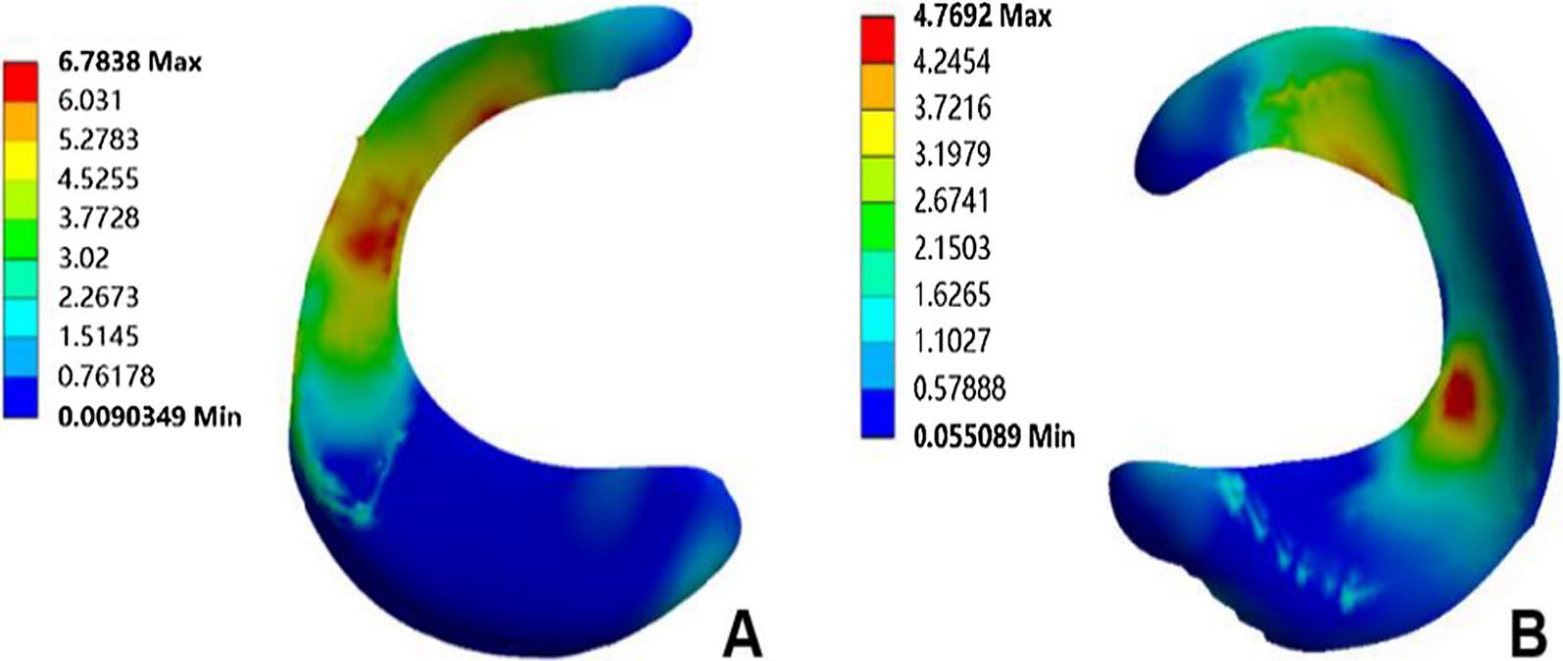
Peak value and distribution of equivalent stress on the surface of medial and lateral meniscus. **A** Medial meniscus, **B** lateral meniscus

The normal knee model could converge after applying the load; the overall number of nodes of the model was 438,170, and the number of units was 273,824. The validation results of method 1 showed that the peak equivalent stresses on the medial and lateral tibial cartilage surfaces were 4.08 MPa and 1.72 MPa, respectively (Fig. 4); the peak equivalent stresses on the medial and lateral femoral cartilage surfaces were 3.84 MPa and 3.31 MPa, respectively (Fig. 5), and the peak equivalent stresses on the medial and lateral meniscus surfaces were 6.78 MPa and 4.77 MPa, respectively (Fig. 6). The loading ratios of the medial and lateral compartments were 58.12% and 41.88%, respectively, both of which were similar to those of previous studies [20–22]. Method 2 validation results showed that the anterior moving distance of the anterior side of the tibial loading point was 4.92 mm, which is consistent with a standard displacement of approximately 5 mm in the anterior drawer experiment, and the results were similar to previous study [21].

This study also observed the stresses in the medial and lateral compartments of the UKA model in the standard position of the femoral prosthesis, which had a loading ratio of approximately 1:2 and a greater peak equivalent force on the upper surface of the PE insert compared to the medial compartment of the regular knee model and more concentrated stress distribution, which is consistent with the results of previous studies [23, 24].

### Stress changes in the internal structure of the knee joint in different UKA models

#### Upper surface of PE insert

**Fig. 7:**
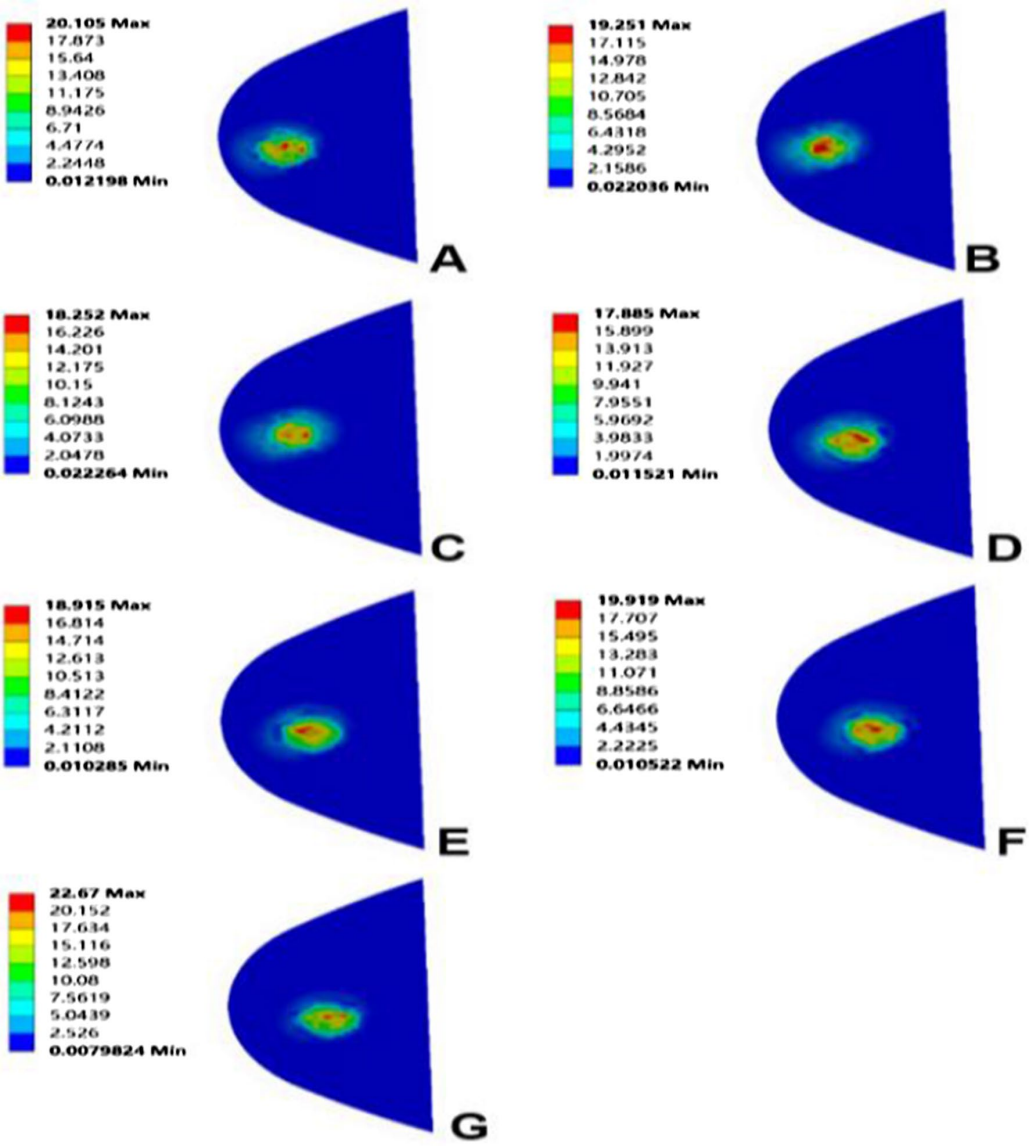
Contact stress distribution on the PE insert with respect to different femoral prosthesis positions in UKA models. **A** 9° varus, **B** 6° varus, **C** 3° varus, **D** standard position, **E** 3° valgus, **F** 6° valgus, **G** 9° valgus

**Fig. 8:**
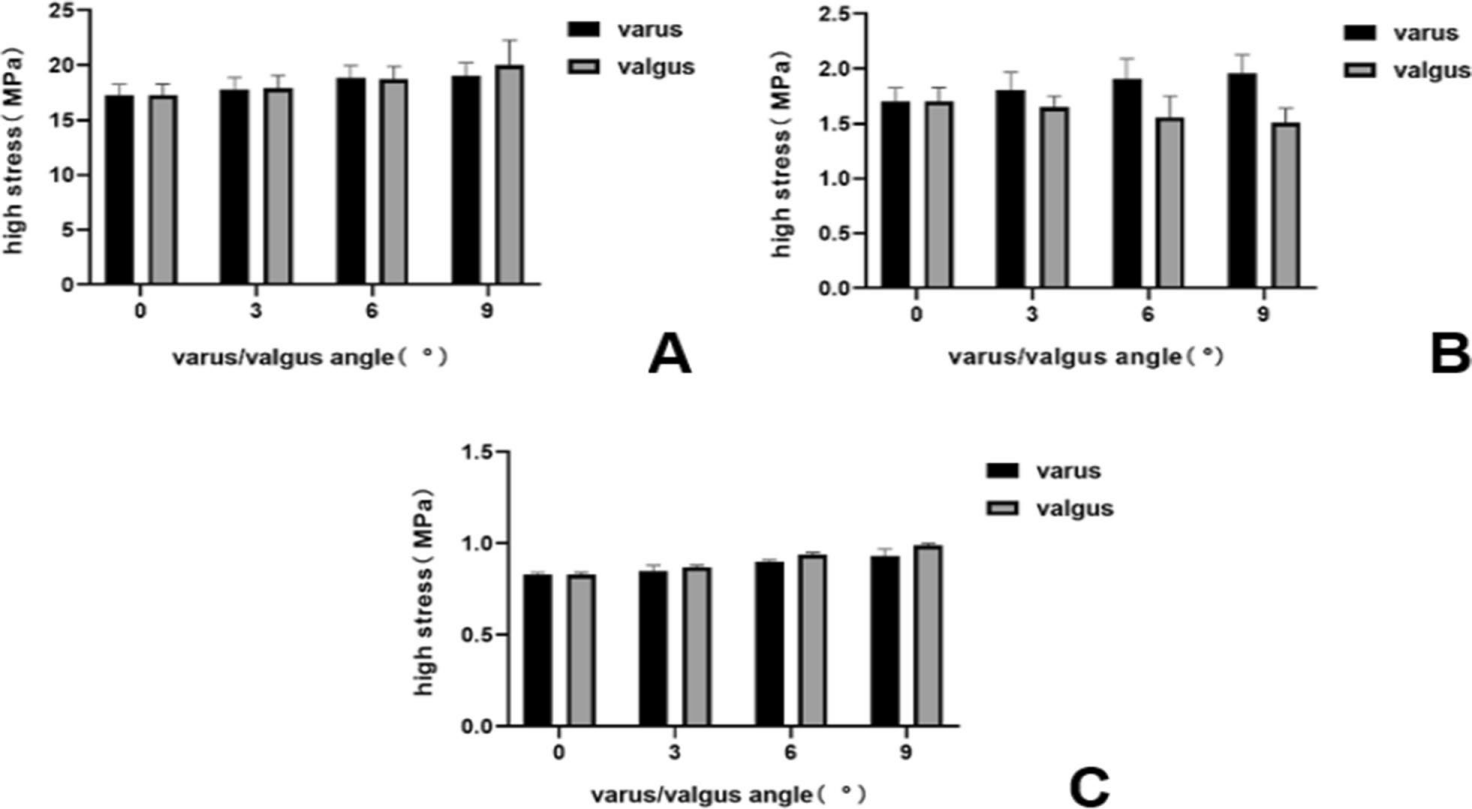
Variation of high-stress in different structures with different femoral prosthesis varus/valgus. **A** Upper surface of PE insert, **B** surface of cartilage in lateral compartment, **C** surface of cancellous bone under tibial prosthesis

**Fig. 9:**
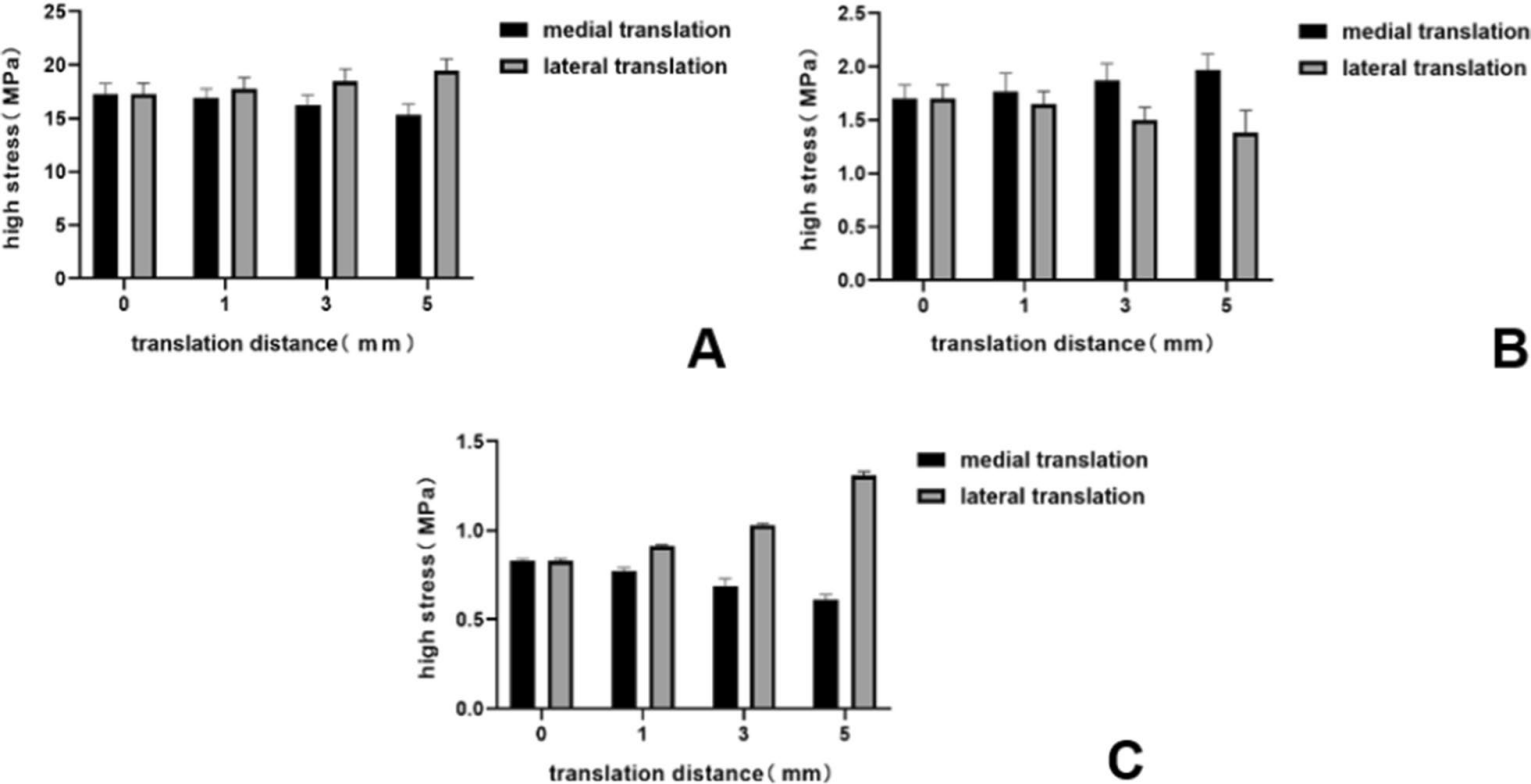
Variation in high-stress in different structures with different femoral prosthesis translations. **A** Upper surface of PE insert, **B** surface of cartilage in lateral compartment, **C** surface of cancellous bone under tibial prosthesis

When the femoral prosthesis is placed in the standard position, the upper surface of the PE presents the lowest high-stress value. In the varus/valgus model, compared with the standard position, statistically significant high-stress values appeared at 6° of the varus/valgus (*P* < 0.05). The peak equivalent stress for the varus and valgus is 20.10 MPa and 22.67 MPa, respectively, both at 9°. The stress increases with the increase of the varus/valgus, and the increase of valgus stress is faster than that of varus (Figs. 7, 8). In the translation model, the stress on the upper surface of the PE increases gradually from 5 mm medial translation to 5 mm lateral translation, and the increase reaches 26%. The increase in stress during lateral translation was greater than the decrease in medial translation of the prosthesis (Fig. 9). Statistical significance appeared when the lateral translation was 5 mm (*P* < 0.05).

#### Surface of cartilage in the lateral compartment

**Fig. 10:**
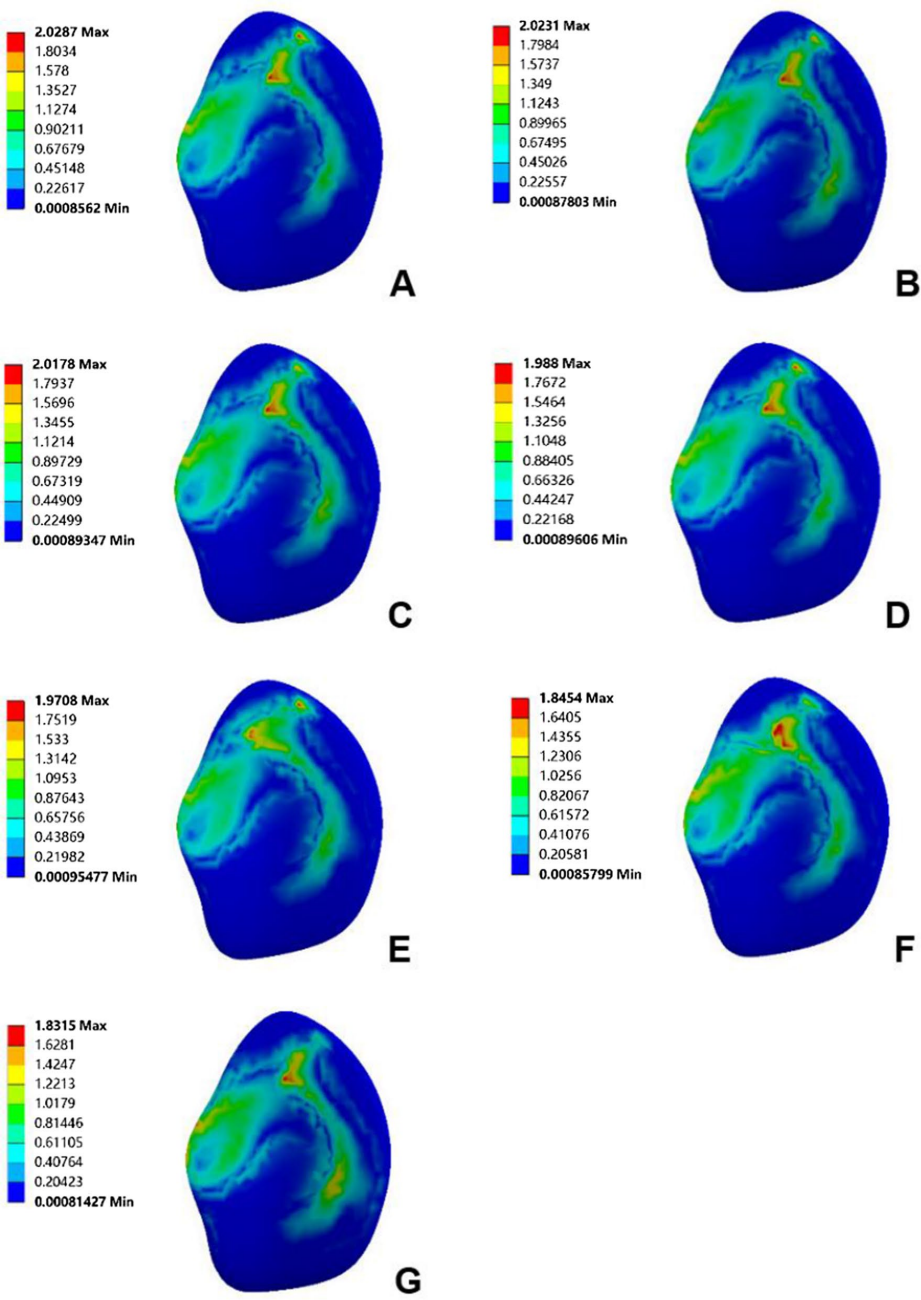
Contact stress distribution on cartilage with respect to different femoral prosthesis positions in UKA models. **A** 9° varus, **B** 6° varus, **C** 3° varus, **D** standard position, **E** 3° valgus, **F** 6° valgus, **G** 9° valgus

Due to the similar variation and trend of the stress in the lateral compartment cartilage, the lateral tibial cartilage is considered representative to describe the stress change. In the varus/valgus model, the high-stress value of cartilage decreases for the valgus and increases for the varus. The stress value increases by 7%, 12% (*P* < 0.05) and 15%, with varus angles of 3°, 6° and 9°, respectively (Figs. 8, 10). In the translation model, the stress change on the surface of cartilage is opposite to that of the PE surface. The results show that from the medial 5 mm to the lateral 5 mm, the stress decreases gradually up to 30%. Similar to the PE insert, lateral translation had a greater impact on the cartilage surface stress, and statistical significance was observed in the medial 5 mm (*P* < 0.05) (Fig. 9).

#### Surface of cancellous bone under tibial prosthesis

There is a ridge in the Link-Sled® tibial prosthesis that fits with the groove on the cancellous bone. The high-stress distribution is located at the bottom and side of the groove in all UKA models. In the varus/valgus model, the high-stress value increases with the increase in varus/valgus, and the stress variation range in valgus is greater than that in varus (Fig. 8). The stress change trends in the translation models are consistent with those of the PE insert. Compared with the standard position of the prosthesis, the stress decreases by 27% when the prosthesis is translated by 5 mm to the medial side, while the stress increases by 58% when it is translated by 5 mm to the lateral side. Compared with other structures, the variation range of cancellous bone surface stress is larger, but the absolute value is smaller (Fig. 9).

## Discussion

UKA is a surgical method that can not only fundamentally relieve the progression of arthritis but also maximize the preservation of bone and soft tissue. However, the revision rate of UKA is higher than that of TKA, which limits the wide applications of UKA [25, 26]. The success of UKA and the reduction of revision rate are strongly dependent on the restoration of alignment, the accurate prosthesis position and the force balance of soft tissue [27]. Some studies have shown that the primary factor that leads to revision is the abnormal contact stress of the articular surface caused by prosthesis malalignment. The excessive stresses on the PE insert and the healthy compartment surface accelerate their wearing and generate particles that cause an inflammatory response that further aggravates the loosening of the prosthesis and the degeneration of cartilage [13, 28]. Thus, a proper and accurate prosthesis position is highly relevant to improve the internal structure stresses of the knee joint and maximize the service life of the prosthesis.

However, at present, the reasonable range of the location of UKA prostheses is still unclear, especially because there are few studies on the biomechanical effects of fixed-bearing prostheses placed in different positions. The clinical duration of fixed-bearing prostheses is relatively short. Moreover, inappropriate operation procedures can lead to prosthesis malalignment [29, 30]. On the other hand, the PE insert of the fixed-bearing prosthesis is immovable so that it is impossible to adjust the abnormal stress caused by prosthesis malalignment. This also reduces the fault tolerance limit of fixed-bearing prostheses to some extent. The edge load of fixed-bearing prostheses is also larger than that of mobile-bearing prostheses, especially in the treatment of the femoral side [31]. Taking the Link-Sled® prosthesis as an example, double-column openings, cartilage grinding files and other procedures often need to be tested repeatedly, as malalignment may affect the life of the prosthesis [10]. In view of the high risk of fixed-bearing femoral prosthesis malalignment during surgery, it is necessary to further analyse its biomechanical characteristics.

Finite element analysis is widely used in the biomechanical study of joints. It can accurately simulate real working conditions, presenting intuitive and accurate results [14]. In the past, finite element analysis for the location of UKA prostheses have mainly focused on mobile-bearing prostheses, which greatly promoted their development [12, 19, 32]. Therefore, in this study, finite element analysis was used to investigate the mechanical effects of the Link-Sled® femoral prosthesis on the internal knee joint structure.

In this study, the results show that the stress on the PE insert is significantly higher than that on the cartilage, which might be attributed to the differences in material parameters between cartilage and PE. The Young's modulus of PE is 56 times higher than that of cartilage, indicating that cartilage can convert more stress into elastic potential energy [24]. For the varus/valgus models, the results show that the high-stress value on the PE insert increases with increasing tilt angle of the femoral prosthesis. This might be because the stress on the PE insert surface is more focused with increasing tilt. The results also show that the high-stress value on the cartilage increases for varus and decreases for valgus tilt. This might be ascribed to the fact that when the femoral prosthesis is tilted, the stress point of PE moves to the medial side and lateral side, resulting in load transfer. Alternatively, this might also be because the medial collateral ligament produces a force to counter the trend during femoral prosthesis tilting [8]. The results also indicate that the trend of stress change on the cancellous bone surface under the tibial prosthesis is consistent with that of the PE insert surface. It should be noted that due to the stress shielding effect and the higher Young's modulus of cortical bone, the stress shared by the cortical bone is much greater than that of cancellous bone [24]. Therefore, it can be assumed that the stress change is caused by the decrease in the stress shared by the cortical bone at the medial edge of the femur, the outward movement of the stress point at the tibial prosthesis and the increased load in the medial compartment of the knee joint.

The results of the femoral prosthesis translation models show a more regular trend. When the femoral prosthesis is translated from the medial side to the lateral side, the stress on the PE insert and the cancellous bone under the tibial prosthesis increases gradually, while the stress on the lateral compartment cartilage surface decreases. The stress variation trend of the medial compartment is completely opposite to that of the lateral compartment. In terms of the mechanical sensitivity to prosthesis malalignment, cancellous bone stress shows obvious changes, followed by the stress of the cartilage and PE insert. However, from the absolute value of stress point of view, the stresses for cancellous bone and cartilage are obviously lower than that of PE insert, which is aligned with the previous study’s findings [33]. Herein, it can be speculated that the medial malalignment of the femoral prosthesis may cause the development of osteoarthritis in the lateral compartment, while lateral malalignment may aggravate the wear of the PE insert and even cause the prosthesis to be loosened [27].

This study provides a significant reference for the clinical use of the Link-Sled® prosthesis on the premise of placing the prosthesis in a relatively safe position and minimizing the poor prognosis caused by the stress imbalance, the force line deviation and the change in effective contact area [34]. A large number of clinical trial results proved the necessity of this analysis. Khow et al. found that patients with a femoral prosthesis varus/valgus greater than 3° presented lower functional scores and poor follow-up results [35]. Another study reported that coronal alignment of the femoral prosthesis is significant for determining the long-term therapeutic benefits of UKA [36].

Once a relatively safe implant range is determined, as demonstrated in this study, appropriate techniques need to be used to accurately position the prosthesis during surgery. Unlike traditional surgical methods, this new technique ensures the accurate reproduction of biomechanical results. For example, the force line position and the planned prosthesis position can be accurately obtained in the applications of computer-assisted technology, including robot-assisted systems and patient-specific instrumentation (PSI), before the operation [37, 38]. Robot-assisted systems require preoperative or intraoperative localization using imaging techniques, matching paired points based on the anatomical structure of the surgical area, and planning osteotomy and prosthesis placement. Finally, the surgeon or robot performs the surgery [39]. Robot-assisted systems can significantly reduce prosthesis positioning error. Cobb et al. compared robot-assisted UKA with traditional UKA, and the results showed that the robot could achieve implant positioning error within 2°, while only 40% of patients in the traditional surgery group showed this result [40]. Aletto et al. [41] performed TKA in 180 patients using an image-free navigation system, and the mechanical axis of all patients was within ± 3° after 2 years of follow-up. As one of the computer-assisted technologies, PSI, like the robotic system, also significantly improves the accuracy of prosthesis alignment. PSI is a guide plate for osteotomy customized for patients by computer technology and 3D printing technology. The anatomical characteristics of the surgical area are determined according to the patient's imaging data during production. The 3D model of the surgical area and PSI is established, and the operation is simulated by referring to relevant guidelines in the software [42]. Bell et al. [37] performed PSI-assisted UKA in 41 patients, and the preoperative plan was accurately achieved in 96% of cases, with a 100% survival rate after 2 years of follow-up. Sanz-ruiz et al. [43] showed that PSI could also improve the alignment accuracy of prostheses for doctors who have less experience with UKA. In addition, new extramedullary devices have been used to improve prosthesis positioning, such as the FuZion® system, which can align the flexion and extension of the knee, achieve mechanical alignment of the lower limb and balance ligament tension. Under the action of ligament tension, the positioning of the femoral prosthesis is more natural, and the tilt of the femoral prosthesis in the coronal plane can be controlled within 5° [44].

It should be emphasized that the innovation of this study lies in the use of more advanced parametric modelling methods. The material parameters and boundary conditions are based on the latest research results. The established finite element models are verified by two methods, which can accurately reflect the biomechanical changes of different working conditions. Unlike previous studies, the models contain complete simulations for bone, cartilage and soft tissue of the knee joint and distinguish cortical bone and cancellous bone [8, 22]. There are limited biomechanical studies investigating the Link-Sled® prosthesis, although it is widely used. This study deepens the understanding of the biomechanical characteristics of this prosthesis. More importantly, this study investigates two groups of models containing the femoral prosthesis varus/valgus tilt and translation, and comprehensively analyses the mechanical effects of femoral prostheses on coronal malposition.

However, there are still some limitations to the current study. Although comparability is ensured between different models considering the data from a single volunteer, the results may lack group representation [45]. In addition, to observe the mechanical effects caused by malposition of the femoral prosthesis on the coronal plane, this study simplifies the bone motion pattern under the force, such as limiting the rotation of the distal femur. Finally, this study adopts the static load in the upright position and lacks the mechanical results of gait cycle simulation under dynamic load.

## Conclusions

Misalignment of the fixed-bearing femur prosthesis during UKA may result in abnormally high stress in the internal structure of the knee. The stress of the PE insert is the greatest and the most concentrated, while the stress on the cancellous bone surface is the least. More attention should be given to reducing the stress on the PE insert and cartilage surfaces during the operation. Coronal plane tilt of the femoral prosthesis over 6° significantly increases the stress on the PE insert, while varus tilt exceeding 6° significantly increases the stress on the cartilage surface. For the femoral prosthesis position at the distal end of the femoral condyle, it is recommended to be placed in the centre. Lateral translation may increase the stress on the PE surface, while medial translation may increase the stress on the cartilage surface.

## Data Availability

The datasets used and/or analyzed during the current study are available from the corresponding author on reasonable request.

## Abbreviations

UKA: Unicompartmental knee arthroplasty
PE: Polyethylene
KOA: Knee osteoarthritis
TKA: Total knee arthroplasty
CT: Computed tomography
MRI: Magnetic resonance imaging
